# Spaceflight Affects Postnatal Development of the Aortic Wall in Rats

**DOI:** 10.1155/2014/490428

**Published:** 2014-08-19

**Authors:** Shin-ichiro Katsuda, Masao Yamasaki, Hidefumi Waki, Masao Miyake, Hirotaka O-ishi, Kiyoaki Katahira, Tadanori Nagayama, Yukako Miyamoto, Masamitsu Hasegawa, Haruyuki Wago, Toshiyasu Okouchi, Tsuyoshi Shimizu

**Affiliations:** ^1^Department of Cellular and Integrative Physiology, Fukushima Medical University, 1 Hikari–ga–oka, Fukushima 960-1295, Japan; ^2^Department of Physiology, Faculty of Clinical Engineering, School of Health Sciences, Fujita Health University, 1-98 Dengakugakubo, Kutsukake-cho, Toyoake, Aichi 470-1192, Japan; ^3^School of Health and Sports Science, Juntendo University, 1-1 Hiragagakuendai, Inzai, Chiba 270-1695, Japan; ^4^Medical Corporations Tenshindo Shida Hospital, 2134-4 Oaza-Nakamura, Kashima, Saga 849-1304, Japan; ^5^Medical-Industrial Translational Research Center, Fukushima Medical University, 1 Hikari–ga–oka, Fukushima 960-1295, Japan; ^6^Nihonmatsu Hospital, 1-553 Narita-cho, Nihonmatsu, Fukushima 964-0871, Japan; ^7^National Cerebral and Cardiovascular Center Research Institute, 5-7-1 Fujishiro-dai, Suita, Osaka 565-0873, Japan; ^8^Shimizu Institute of Space Physiology, Suwa Maternity Clinic, 112-13 Shimosuwa, Suwa-gun, Nagano 393-0077, Japan

## Abstract

We investigated effect of microgravity environment during spaceflight on postnatal development of the rheological properties of the aorta in rats. The neonate rats were randomly divided at 7 days of age into the spaceflight, asynchronous ground control, and vivarium control groups (8 pups for one dam). The spaceflight group rats at 9 days of age were exposed to microgravity environment for 16 days. A longitudinal wall strip of the proximal descending thoracic aorta was subjected to stress-strain and stress-relaxation tests. Wall tensile force was significantly smaller in the spaceflight group than in the two control groups, whereas there were no significant differences in wall stress or incremental elastic modulus at each strain among the three groups. Wall thickness and number of smooth muscle fibers were significantly smaller in the spaceflight group than in the two control groups, but there were no significant differences in amounts of either the elastin or collagen fibers among the three groups. The decreased thickness was mainly caused by the decreased number of smooth muscle cells. Plastic deformation was observed only in the spaceflight group in the stress-strain test. A microgravity environment during spaceflight could affect postnatal development of the morphological and rheological properties of the aorta.

## 1. Introduction

It is well known that blood shifts headward immediately after exposure to a microgravity (*μ*G) environment and thereafter decreases in volume to adapt to the environment, which could affect cardiovascular hemodynamics and associated regulatory mechanisms [1, 2]. Central venous pressure (CVP) [3, 4], cardiac output (CO) [5, 6], and arterial pressure (AP) [7, 8] have been reported to instantaneously increase after exposure to *μ*G and then decrease in the process of adapting to *μ*G environment during spaceflight in humans.

On the other hand, cardiovascular function changes concomitant with growth after the birth [9]. Blood pressure has been shown to gradually elevate to almost the mature level by the age of 8 weeks [10] or 45 days [11] in Sprague-Dawley (SD) rats and at 4 weeks of age in Wistar Kyoto rats [12]. Baroreceptor sensitivity has also been reported to develop with growth [10, 11]. AP is determined by the rheological properties of the aortic wall as well as by cardiovascular hemodynamics. Baroreflex function is susceptible to the rheological properties of the aortic wall in which they lie [13, 14]. The rheological properties are closely related to alteration in the fine structure of the wall [15–20]. Baroreceptor function and rheological properties of the aortic wall are considered to develop with morphological growth of the heart, blood vessels, and other cardiovascular components. In our research group, postnatal development of the baroreflex system has been studied under ordinary gravitational conditions [9] and simulated microgravity conditions such as head-down tilt (HDT) [21, 22] and parabolic flight [22, 23]. Yamasaki and Shimizu [24] showed previously in 3-4-week-old rabbits raised in HDT posture in a simulated *μ*G environment for 34–36 days that the number of unmyelinated fibers of the left aortic nerve was significantly reduced compared to the control rabbits, which suggested that development of the aortic baroreflex sensitivity was depressed by exposure to HDT posture. It is thus possible that a similar phenomenon is observed during spaceflight in neonate animals. Therefore, we suspected that the rheological and histological properties of the aortic wall as well as cardiovascular hemodynamics could be modulated by exposure to a *μ*G environment during growth and investigated to verify the hypothesis in the NASA Neurolab Programs (STS-90) [25] where we joined with the theme “development of the aortic baroreflex in microgravity.”

## 2. Materials and Methods

### 2.1. Animals and Animal Care

**Figure 1:**
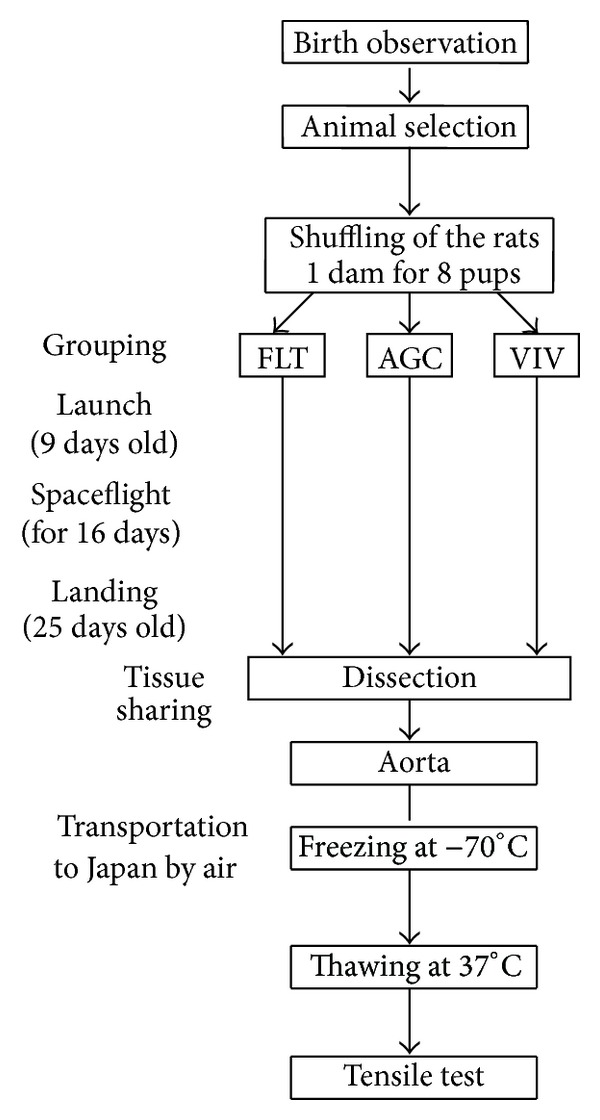
Flowchart of the experiment from the birth of rats to the tensile test. FLT: spaceflight, AGC: asynchronous ground control, and VIV: vivarium control.

Figure 1 shows a flowchart of the period from the birth to tensile test of the rats after the spaceflight. Eight neonate Sprague-Dawley rats in a good state of health and development, selected from a large colony 5 days after birth, were randomly assigned to one mother rat as one litter. A total of 18 litters were randomly and equally divided into three groups at 7 days after the birth: the spaceflight (FLT), asynchronous ground control (AGC), and vivarium control (VIV) groups. One litter out of the 6 litters in each group was assigned for the present study. The FLT group rats were bred in the specially designed Research Animal Holding Facility (RAHF) [26] loaded on board of the Space Shuttle. The RAHF cage is 4.00 × 4.25 × 10.00 inches and can accommodate one dam and eight pups. The AGC and VIV group rats were housed in simulated RAHF and standard commercial (18.50 × 10.25 × 8.50 inches) cages, respectively, under one-G conditions and the same temperature (23 ± 1°C) and light and dark cycle as the FLT group rats. All rats were given SLO Foodbars and were cared for by a veterinarian crewmember during spaceflight and specialized personnel before and after the flight. The FLT group rats were exposed to a *μ*G environment in the Space Shuttle “Columbia” for 16 days from 9 to 25 days after the birth. The FLT group rats were examined for basic health conditions immediately after landing and then dissected within 10 hrs after sampling blood under pentobarbital anesthesia (50 mg/kg body weight, i.p.). The tissues and organs were shared among some research teams joined to the Neurolab Programs (STS-90). In the AGC and VIV group rats, the same experimental procedures were employed, except for breeding under *μ*G conditions. The aorta was excised from the origin of the ascending aorta to the thoracic aorta, gradually frozen to −70°C, transported to Japan by air, and stored at −85°C to minimize damage due to freezing. All experimental procedures were performed according to the guidelines for Animal Care and Use in NASA and NIH.

### 2.2. Tensile Test

Prior to the tensile test, we investigated the differences in the tensile characteristics between the fresh and thawed proximal descending thoracic aorta in premature rats aged 3 weeks. There were no observable differences in the tension-strain or stress-strain relations between the fresh and thawed rat aorta (Katsuda and Hasegawa unpublished observations). The experimental procedure was similar to that described previously [19, 20, 27]. The proximal descending thoracic aorta was cut from the bifurcation of the left subclavian artery to the third intercostal arteries and cut longitudinally into 3 mm wide strips after rapid thawing to 37°C. The rheological properties of the strips were measured by a tensile testing instrument (TOM-30J, Minebea, Inc., Japan) which mainly consists of a load cell, a movable crosshead, a driving unit, and a chamber [19]. One end of the strip was mounted between the jaws of a chuck and it was suspended on a load cell with a flexible wire. Another end was held by another chuck attached to the organ bath of the tensile testing instrument. The sample was immersed in saline solution consisting of NaCl (147.2), KCl (2.7), MgCl_2_ (0.5), CaCl_2_ (1.8), NaH_2_PO_4_ (1.0), Na_2_HPO_4_ (3.0), and glucose (5.6) (mM) at 37°C. Initially, the strip was held at the maximum length where the tension just exceeded 0 N. After holding at the initial length, the strip was subjected to force-strain test. The tensile force in the sample was generated by mechanical stretching to about 1 N at a speed of 4.2 mm/min, relaxed to the initial length immediately after the stretching, and kept relaxed for 5 min. After plastic deformation was measured at 5 min after the relaxation of the strip, the strip was subjected to a stress-relaxation test. The strip was stretched by 50% of the initial length at a speed of 83.3 mm/min and sustained for 5 min. Immediately after the test, the strip was cut off at the margin of each chuck and weighed on a precision balance. Strain of the wall strip (*ε*) was defined as *ε* = (Δ + *L*_0_)/*L*_0_, where *L*_0_ and Δ were initial length of the strip and increment from the initial length, respectively. The stress value (*σ*) at any moment during the stretching was determined using the following formula: *σ* = 1.06 × *L*_0_(1 + *ε*) × *T*/*W*, where *T* was the tension (g) of the strip, *W* the sample weight, *L*_0_ initial length of the strip (cm), and *ε* strain of the strip. Poisson's ratio and density of the aortic wall were assumed to be 0.50 [28] and 1.06 g/cm^3^ [29], respectively. The incremental elastic moduli of the wall (*E*) at strain levels of 0.25, 0.50, and 0.75 with respect to the unstressed length were selected as the mean gradient of the stress-strain curve at strains between 0.20 and 0.30, between 0.45 and 0.55, and between 0.70 and 0.80, respectively. For example, the value of *E* at the strain of 0.5 was expressed as (*σ*_0.55_ − *σ*_0.45_)/(*ε*_0.55_ − *ε*_0.45_), where *σ*_0.55  _ and *σ*_0.45_ were stress at strains of 0.55 and 0.45 and *ε*_0.55_ − *ε*_0.45_ difference in strains (e.g., 0.1), respectively. The relaxation strength was calculated by (*τ*_0_ − *τ*_5min⁡_)/*τ*_0_ × 100 (%), where *τ*_0_ was the maximal tension generated immediately after stretching and *τ*_5min⁡_ the tension at 5 min after the stretching (Figure 4). The plastic deformation of the strip was measured at 5 min after the relaxation of the sample in the stress-strain test. Wall thickness of the strip (*h*) was calculated as *h* = *W*/(1.06 × *L*_0_ × *Wd*), where *Wd* was the width of the sample (cm). Internal radius of the descending proximal thoracic aorta was estimated as *l*/2*π*, where *l* was the circumferential length of the excised wall strip.

### 2.3. Histological Sections

The strips were fixed in 10% neutral buffered formalin solution and embedded in paraffin. Circumferential and longitudinal histological sections were sliced at 5 *μ*m thickness and stained with Elastica-van Gieson (EVG) and hematoxylin-eosin (HE).

### 2.4. Image Analysis

The images of smooth muscle cells (SMC), elastin fiber, and collagen fiber in the longitudinal histological sections stained with EVG, which were displayed at yellow, black, and red, respectively, were sampled by an image analysis system (LUZEX FS, Nireco Corporation, Tokyo, Japan) through a microscope (Olympus BX-50, Olympus Corporation, Tokyo, Japan) at a magnification of 40 times and a CCD-video camera operated by a camera control unit. The image within a frame of an image analysis system (2.52 × 10^−4^ *μ*m^2^ in area) was converted to the sliced video images prior to processing by the main processor. An outline image of each element was discriminated by the adjusting intensity, hue, and purity of its color and was selectively extracted. The three components and the entire sectional area were binarized and the intensity and tint were adjusted to the background. The SMC, elastin fiber, collagen fiber, and entire sectional area were measured with the main processor. The area of each component was expressed as a percentage of the entire sectional area in each histological section. These procedures for analyzing the three major components were repeated in at least two microscopic fields of each histological section. The image of the SMC stained with HE was taken into an image analysis system and binarized in a similar way to images of the longitudinal sections stained with EVG at a magnification of 40 times. The outline image of the nucleus in a given frame area (2.52 × 10^−4^ *μ*m^2^ in area) was emphasized for discrimination by adjusting the intensity, hue, and purity of its color, displayed in blue, and selectively extracted. The number of nuclei seen within one frame for one section stained with HE system was counted using an image analysis system. These procedures were repeated in three microscopic fields for each histological section. The number of nuclei in three microscopic fields was averaged within each rat group.

### 2.5. Statistical Analysis

The experimental data, for example, FLT versus ACG, FLT versus VIV, and ACG versus VIV, were compared by Scheffe's multiple comparison tests after confirming significant differences by one-way analysis of variance (ANOVA).

## 3. Results

Total number of pups available for all areas of research decreased after the landing of Space Shuttle, so that we were consequently forced to reduce the number of pups for a series of experiments. Six pups were ultimately allotted to the FLT, AGC, and VIV groups in the present study, respectively, after number of pups had been readjusted to share as fairly as possible.

Table 1 summarizes body weight and physical characteristics of the proximal thoracic descending aorta. Internal diameter was estimated from the excised strip of the proximal thoracic aorta. Body weight in the FLT group was about half of that in the two control groups and significantly lower than that in the AGC (*P* < 0.001) and VIV (*P* < 0.001) groups. Weight of the proximal descending thoracic aorta per unit area (cm^2^) tended to be small in the FLT group compared with that in the two control groups, which was not statistically significant. Cross-sectional area of the aortic wall in the FLT group was significantly small compared with that in the AGC (*P* < 0.01) and VIV (*P* < 0.01) groups. Internal diameter was significantly smaller in the FLT group than in the AGC (*P* < 0.001) and VIV (*P* < 0.01) groups.

**Figure 2:**
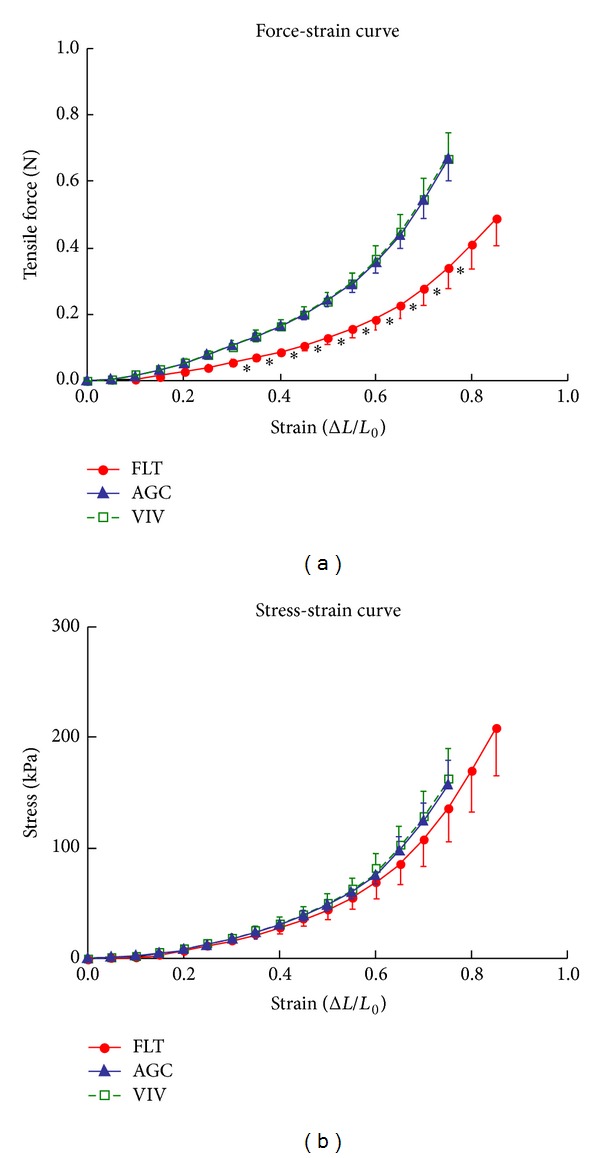
Force-strain (a) and stress-strain (b) curves of the longitudinal strips excised from the proximal thoracic aorta in the FLT, AGC, and VIV group rats. Values are mean ± SE. *:*P* < 0.05 (FLT versus AGC and FLT versus VIV), *L*_0_: initial length of the strip, and Δ*L*: increment by stretching.

**Figure 3:**
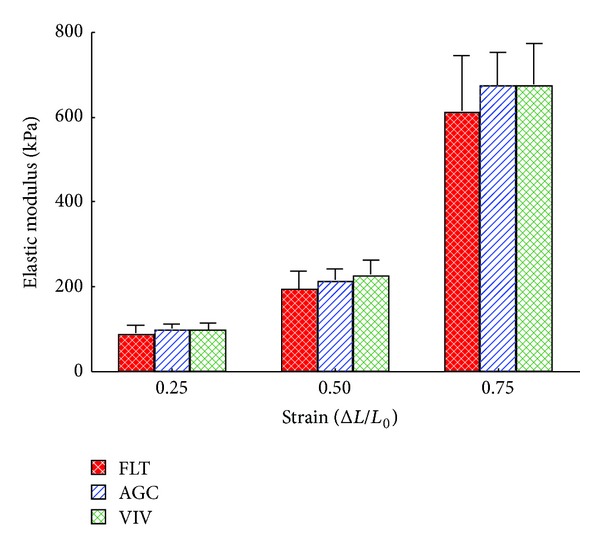
Incremental elastic modulus of the longitudinal strips excised from the proximal thoracic aorta in the FLT, AGC, and VIV group rats. Values are mean ± SE. Abbreviations are similar to those in Figure 2. Incremental elastic modulus was determined at strains of 0.25, 0.50, and 0.75.

**Figure 4:**
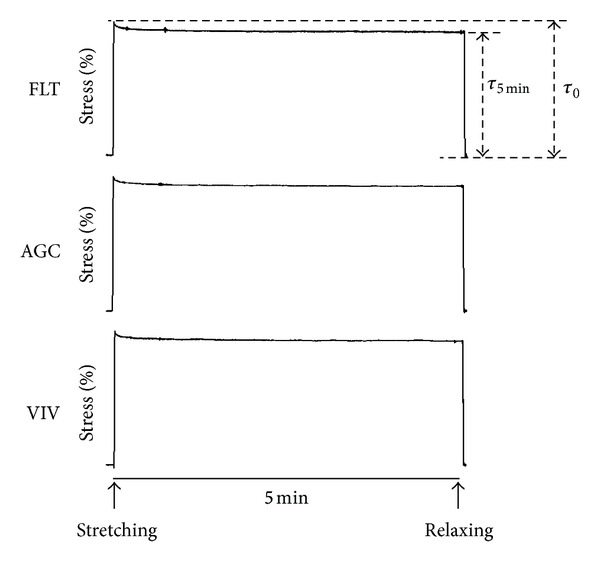
Examples of stress-relaxation of the longitudinal strips excised from the proximal thoracic aorta in the FLT, AGC, and VIV group rats. The strips were stretched by 50% from the initial length. Relaxation strength was defined as a percent ratio of *τ*_0_/(*τ*_0_ − *τ*_5min⁡_), where *τ*_0_ was peak stress immediately after the stretching and *τ*_5min⁡_ stress at 5 min after the stretching.

**Figure 5:**
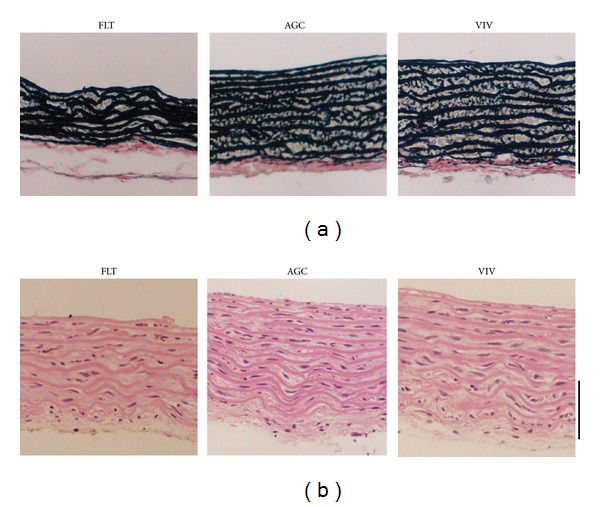
Photomicrographs of the longitudinal (a) and circumferential (b) histological sections of the proximal descending thoracic aorta stained with EVG (a) and HE (b) stains in the FLT, AGC, and VIV group rats. Perpendicular bar: 100 *μ*m.

**Figure 6:**
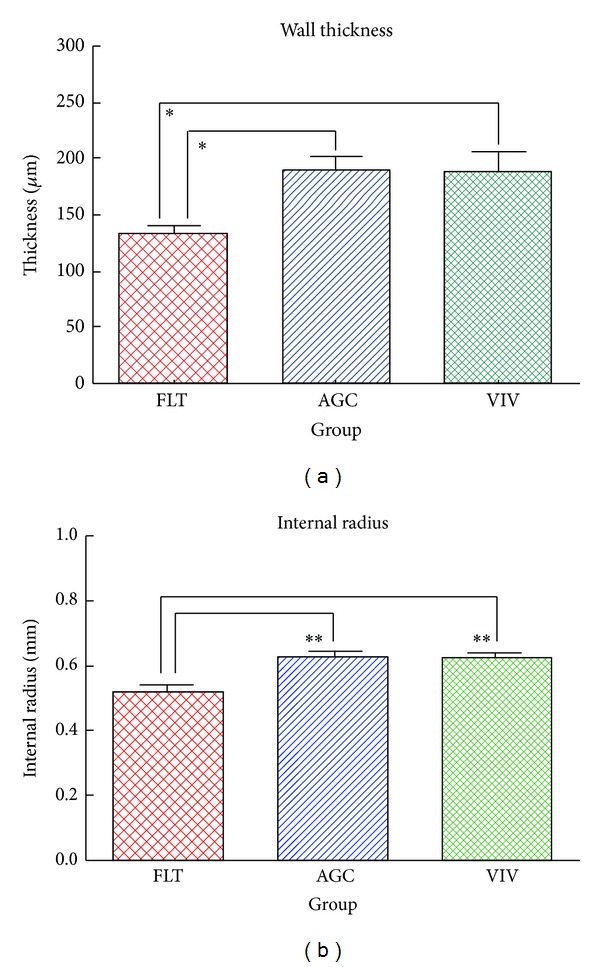
Estimated wall thickness (a) and internal radius (b) of the proximal thoracic aorta in the FLT, AGC, and VIV group rats. Values are mean ± SE. *P < 0.05, **P < 0.01.

**Figure 7:**
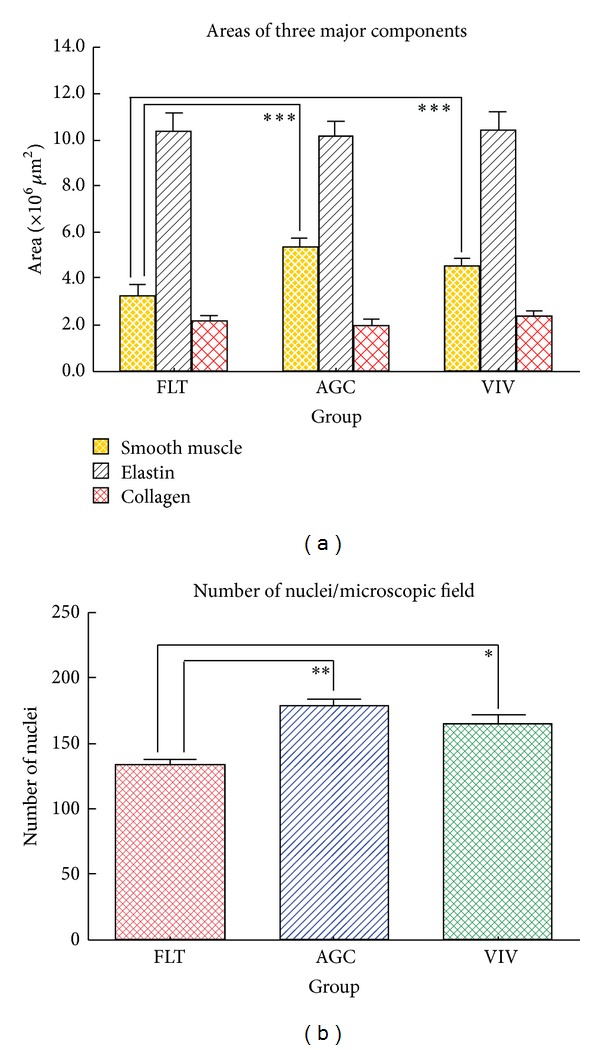
Area of the smooth muscle, elastin, and collagen fibers (a) in the longitudinal histological sections and number of nuclei of the smooth muscle cells (b) in the circumferential histological sections excised from the proximal thoracic aorta in the FLT, AGC, and VIV group rats. Values are mean ± SE. *P < 0.05. **P < 0.01. ***P < 0.001. Area of each component was measured in three given microscopic fields for one histological section stained with EVG with an image analysis system and then averaged within each group. The number of nuclei was measured in three given microscopic fields for one section stained with HE with an image analysis system and then averaged within each rat group.

Figure 2(a) shows the force-strain curves in the longitudinal strips excised from the descending proximal thoracic aorta in the FLT, AGC, and VIV group rats. As the strain increased, the tensile force gradually elevated in the three groups. The difference in tensile force between the FLT and the two control groups gradually widened as strain increased. The tensile force in the FLT group rats was significantly smaller than those in the AGC and VIV group rats at a strain range between 0.30 and 0.75 (*P* < 0.05).  Figure 2(b) illustrates stress-strain curves derived from the corresponding force-strain curves in the three groups. The contour of the stress-strain curve resembles that of the force-strain curve. There were no significant differences in stress value between any two groups at any strain value (*P* > 0.05). The values of *E* at strains of 0.25, 0.50, and 0.75, which correspond to low, medium, and high physiological strain values of the aorta, respectively, are depicted in Figure 3. The value of *E* was about 100 kPa at a strain of 0.25, nearly doubled at a strain of 0.50, and at 0.75 drastically increased to about three times the strain at 0.50 in the three groups. There was no significant difference in the value of *E* between any two groups at any strain (*P* > 0.05). Figure 4 shows examples of stress-relaxation curves in FLT, AGC, and VIV rats. The pattern of the curve was almost similar among the three groups. Relaxation strength at 5 min after 50% stretching beyond the initial length in the FLT, AGC, and VIV groups (*n* = 6 in each group, mean ± SE) was 8.4 ± 1.7, 7.7 ± 0.9, and 7.6 ± 1.0 (%), respectively, which showed almost the same value (about 8.0%) in the three groups and was not significantly different between any two groups (*P* > 0.05). Plastic deformation of the strip measured at 5 min after the relaxation following the stress-strain test was observed in all the strips of the FLT group (0.12 ± 0.03 mm, mean ± SE) only despite showing no significant difference in the value of *E* compared to the two control groups, whereas plastic deformation was not detected in the two control groups. Figures 5(a) and 5(b) are photomicrographs of the longitudinal and circumferential histological sections of the proximal descending thoracic aorta stained with EVG and HE stain in the three groups, respectively. It is interesting that the smooth muscle layer in the FLT group was thin compared to that in the two control groups. The thick elastin fibers in the FLT group were almost the same in number, thickness, and amount as in the two control groups. The fine elastin fibers connecting the thick elastin fibers and smooth muscle cells to each other were circumferentially and longitudinally fast woven in the two control groups, whereas they were poorer in number and networking in the FLT group than in the control groups. The collagen fibers were also similar in amount and arrangement among the three groups, although the wall was considerably compressed in the FLT group rats. The number of nuclei in the smooth muscle cells was considerably smaller in the FLT group than in the two control groups. No histological alteration in the smooth muscle cells, for example, change in size or shape, was clearly detected by microscopic observation. Figures 6(a) and 6(b) illustrate estimated wall thickness and internal radius of the proximal descending thoracic aorta in FLT, AGC, and VIV groups. Wall thickness in the FLT group was 133.3 ± 17.8 *μ*m (mean ± SE) and significantly decreased to about 70% of that in the two control groups (193.4 ± 11.5 for AGC group, *P* < 0.05, and 188.4 ± 18.2 *μ*m for VIV group, *P* < 0.05). Internal radius was significantly smaller in the FLT group rats than that in the AGC and VIV group rats.  Figure 7(a) illustrates the areas of elastin and collagen fibers and smooth muscle cells as measured in the longitudinal histological sections in the three groups using an image analysis system. The area of smooth muscle in the longitudinal histological section was significantly smaller in the FLT group than in the AGC (*P* < 0.001) and VIV (*P* < 0.001) groups, whereas the areas of the elastin and collagen fibers were not significantly different between any two groups (*P* > 0.05). The number of nuclei in one microscopic field in the image analysis system was 134.3  ±  3.6, 179.3 ± 5.5, and 166.2  ±  5.2 (mean ± SE) in the FLT, AGC, and VIV groups, respectively (Figure 7(b)). The number of nuclei was significantly smaller in the FLT group than in the AGC (*P* < 0.01) and VIV (*P* < 0.05) groups, respectively.

## 4. Discussion

The extracellular fluid first shifts headward in exposure to *μ*G environment and decreases in volume in the course of acclimatizing to the environment. The decrease in fluid volume induced a decrease in CO, which could partly involve lowering of blood pressure [1, 2]. CO has been demonstrated to reduce by approximately 15% from the preflight level in astronauts during sustained spaceflight [5, 6]. Fritsch-Yelle et al. [7] reported that diastolic pressure and heart rate significantly decreased and that systolic pressure tended to fall during spaceflight in humans. Gazenko et al. [8] also observed the decrease in diastolic pressure in humans during spaceflight.

AP could be estimated by the rheological properties of the wall as well as by the parameters of cardiovascular hemodynamics such as cardiac output and peripheral vascular resistance. AP can theoretically be expressed by Laplace's law, for example, AP = *T*/*R* = *Ehε*/*R*, where *T* is tension of the wall, *E* elastic modulus of the wall, *h* thickness of the wall, *ε* strain of the wall, and *R* radius of the blood vessel. In our other analysis of cardiovascular function in Neurolab Program (STS-90), mean arterial pressure (MAP) measured about 12 hrs after the landing of the Space Shuttle was significantly lower in the FLT group rats than in the other AGC and VIV group rats, respectively [30, 31]. The time lag between the landing and arterial pressure measurement seems insufficient to adapt completely to the one-gravity (one-G) environment. There was no significant difference in the values of *E* among the FLT, AGC, and VIV groups, respectively, although it tended to show a slight decrease in the FLT group. The significantly decreased wall tension in the FLT group mainly due to the reduction in wall thickness could partly be responsible for the fall in MAP level in the FLT group during the spaceflight.

The aortic wall consists in major part of the elastin and collagen fibers and SMC, whose content and arrangement have been morphologically demonstrated by optical and electron microscopic studies [16, 32]. The elastin fibers form a robust network in the proximal aortic region, while the network becomes sparser with increasing longitudinal cracks in the distal region of the aorta [19, 20]. The collagen fibers also show strong network structure in all aortic regions, though they were crimped or relaxed in an ordinarily stretched state [19, 20]. SMC are arranged in a spiral or helical manner. The number of turns of SMC spirals for a given length increases and the angle between the spiral plane and the transactional plane decreases with increasing distance from the heart [19, 20]. The elastin fiber and SMC are considered to contribute in major part to the elastic and viscous properties, respectively, of the aorta within the normal range of arterial pressure. The collagen fibers are thought to protect the aortic wall from rupturing when exposed to abnormally high pressure [15, 33].

The static rheological properties of the aortic wall have been shown to differ by arterial segment, direction of the wall, age, species, and other factors [34–40]. Azuma and Hasegawa [19, 20] previously investigated the difference in the rheological properties of the aorta between circumferential and longitudinal directions. The static rheological characteristics of the aortic wall become gradually viscoelastic in the circumferential direction with increasing distance from the heart, while they were elastic in the longitudinal direction irrespective of the portion of the aortic tree. There was no marked difference in the viscoelastic properties between the circumferential and longitudinal directions in the proximal aortic region. These mean that the proximal aorta behaves as an elastic vessel to achieve auxiliary pumping function.

In the present study, wall stress did not differ at any strain level between any two groups, though wall tensile force was significantly smaller in the FLT group than in the two control groups. This was caused by the significant decrease in wall thickness in the FLT group, which was considered to be chiefly due to the reduction in the amount of SMC. The number of SMC nuclei was significantly decreased in the FLT group in comparison to the two control groups, which contributed to the decrease in the SMC layer. Other important factors affecting the thickness of the SMC layer were the size of the SMC and the volume of the extracellular fluid surrounding the SMC. It is not plausible that a large amount of intracellular fluid was deprived to alter the contour of the SMC in the *μ*G environment because severe dehydration in neonate rats over the period of lactation would not allow maintaining their lives in space. The size of the SMC may be well preserved; however, we did not investigate the size with an electron microscope in the present study. A decrease in the extracellular volume during spaceflight would partly contribute to the decrease in the SMC layer in addition to the reduction in the number of the SMC. The significant decrease in the internal diameter of the aorta and body weight in the FLT group rats would partly support a decrease in the extracellular volume during spaceflight. The decreasing trend of the weight of the aorta and the significant decrease in cross-sectional area of the aorta in the FLT rats are considered to mainly reflect the decreased SMC layer. The lower mass of the aortic wall possibly affects the rheological properties of the aortic wall through the decreased SMC layer in FLT rats. It is unlikely that the lower mass of the aorta per se alters overall rheological characteristics of the aortic wall in the FLT group because the elastin and collagen contents were almost the same as those in the two control groups.

The relaxation strength was about 8% in the three groups, which suggested that the wall in the proximal thoracic aorta was almost elastic regardless of the presence or absence of spaceflight. The elastic properties would reflect the histological findings that there were no significant differences in the elastin and collagen fiber content and that the arrangement of thick elastin fibers was not markedly altered in any of the three groups. The plastic deformation was observed in all strips in the FLT group, whereas it was not detected in the two control groups. This was likely caused in part by the decrease in the elastic recoil due to the insufficient formation of the fine elastin fibers connecting the thick elastin fibers or SMC.

Cardiovascular function and rheological properties of the aortic wall are known to gradually develop with growth after birth [9–12, 24, 27]. Waki et al. [10] investigated changes in the MAP level and baroreceptor function with postnatal development in SD rats at 3, 8, and 20 weeks of age and reported that MAP level reached mature level by 8 weeks, although the sensitivity of baroreceptors was significantly smaller at 3 and 8 weeks than at 20 weeks. Dickhout and Lee [12] showed an increase in MAP level with postnatal development and reaching approximately mature level at 4 weeks of age in Wistar Kyoto rats. Kasparov and Paton [11] also reported progressive increase in MAP level with growth from 6 to 25 days after birth. We previously observed that the value of relaxation strength at 5 min after the stretching was 9.3%, 8.2%, and 4.6% on average in SD rats at 3, 8, and 20 weeks of age, respectively, and that wall tensile force at a given strain and thickness gradually increased with growth by the age of 20 weeks [27]. These support strongly that cardiovascular function and rheological properties are still in development at least at 3 weeks of age.

An important question arises whether breeding in *μ*G environment affects growth or not. Nutritional state could have significant impact on growth. In the present study, the body weight in the FLT rats was significantly low compared with that in the two control groups on the day of the landing on the ground. The most likely cause seemed to be lack of nursing due to a reduced interaction between the dam and pups during spaceflight. However, it was extraordinarily difficult for the astronauts to measure daily milk intake and body weight in a confined cabin of the Space Shuttle under microgravity conditions. They could only check physical conditions of rats by appearance in the *μ*G environment. However, some scientific bases have been shown that nutritional disturbance during spaceflight was not a major cause of the morphological and functional alterations in the FLT rats. Walton et al. [41] showed that the body weight in the FLT rats reached that of the AGC rats and VIV rats by 8 and 13 days after the landing, respectively. Adams et al. [42] demonstrated that the myosin heavy chain (MHC) genes, very sensitive to lack of nutrition, were not expressed in the FLT rats in the Neurolab experiment (STS-90), whereas expression of cardiac *β*-MHC was predominant in malnourished status. We investigated the rheological properties in the same pups as those shared with the team examined MHC gene expression. Oishi et al. [43] reported that the aortic ring in the FLT rats showed smaller or no vasoconstriction response to phenylephrine compared with that in the AGC and VIV rats. Furthermore, the phenylephrine-induced vasoconstriction response in the lactation-restricted rats little changed compared with that in the normal rats. Stein et al. [44] compared morphological findings of SMC of the aorta using an electron microscope in growth-arrested rats at 18 and 33 weeks of age by either inhibition of thyroid function or caloric restriction at 5 weeks with those in age- and body-weight-matched controls. They reported that the ultrastructural appearance was similar among these groups, though aortic weight, DNA, cholesterol, and phospholipid contents differed. We previously investigated the effects of malnutrition on the rheological characteristics of the proximal descending aorta in 16-day-old neonate rats restricted from nursing for 9 days (Katsuda et al. unpublished observations). There were no significant differences in wall tensile force, wall stress, incremental elastic modulus, wall thickness, or number of nuclei in the SMC between the nursing-restricted and control rat groups, although body weight was significantly smaller in the suckling-restricted groups than that in the control groups. It cannot be denied that the *μ*G conditions affect the morphology and function of the vascular system, although detailed study on nutritional matter in the *μ*G environment is required in the future.

Most investigators have reported the *μ*G environment could affect growth of the nervous and muscular system in neonate rats in the Neurolab study (STS-90) [31, 41, 42, 45, 46]. Adams et al. [45] demonstrated that the growth of body and limb skeletal muscles of neonate rats was impaired under *μ*G environment and that systemic and body expression of insulin-like growth factor-I (IGF-I) was suppressed by spaceflight for 16 days. Yamasaki et al. [46] reported that the number of high-threshold unmyelinated fibers of the aortic nerve was significantly smaller in the neonate rats exposed to *μ*G environment for 16 days than in the control rats bred under one-G conditions. Waki et al. [31] also demonstrated that the baroreflex function in neonate rats was attenuated at 12 hrs after the returning from the spaceflight for 16 days. In the present study, the diameter of the aorta, the wall thickness, and the number of SMC in the proximal thoracic aortic wall were significantly reduced in the FLT group compared to those in the control groups. Morphological and rheological properties are considered to be affected by exposure to a *μ*G environment in the course of development. The neonate rat might not need to develop the aortic wall thickness, internal diameter, and strength much to increase blood pressure and to pump out a large amount of blood toward peripherals under *μ*G conditions because the rat would not fully move muscles against gravity in the process of growth during spaceflight.

In conclusion, the *μ*G environment in the space could affect the morphological and rheological properties of the aorta in the process of growth in neonate rats. The present study offers fundamental data on vascular physiology and morphology in animals and humans for long-term stay in space station.

## Figures and Tables

**Table 1:** Body weight and estimated weight and internal diameter of the proximal descending thoracic aorta in the FLT, AGC, and VIV group rats.

	Pups number	#1	#2	#3	#4	#5	#6	Mean	SE
Body weight (g)	FLT	54.1	31.1	24.5	32.7	43.7	53.9	40.0^∗∗∗a,b^	5.1
	AGC	83.0	78.8	71.2	71.4	78.7	81.9	77.5	2.1
	VIV	87.4	80.3	81.4	88.8	80.9	78.9	83.0	1.7

Weight of the aorta per cm^2^ (mg)	FLT	14.2	16.0	13.9	16.4	13.6	10.9	14.2	0.8
	AGC	17.1	16.5	23.3	24.1	22.4	16.4	20.0	1.5
	VIV	19.5	14.0	14.3	26.4	24.9	20.0	19.9	2.1

Cross-sectional area (mm^2^)	FLT	0.51	0.46	0.40	0.51	0.46	0.37	0.45^∗∗a,∗∗b^	0.02
	AGC	0.69	0.65	0.84	0.93	0.88	0.68	0.78	0.05
	VIV	0.69	0.58	0.57	1.12	0.91	0.74	0.77	0.09

Internal diameter (mm)	FLT	1.20	0.97	0.97	1.05	1.13	1.17	1.08^∗∗∗a,∗∗b^	0.04
	AGC	1.37	1.33	1.22	1.30	1.32	1.38	1.32	0.02
	VIV	1.19	1.39	1.35	1.43	1.24	1.26	1.31	0.04

**P < 0.01, ***P < 0.001, ^a^FLT versus AGC, and ^b^FLT versus VIV. Body weight was measured on the day of the landing. Internal diameter was estimated from circumferential width of the wall strip excised from the proximal descending thoracic aorta. The cross-sectional area was calculated from width and thickness of the wall strip. The diameter is expected to be stretched approximately 50% in situ.

## References

[1] Hargens AR, Watenpaugh DE (1996). Cardiovascular adaptation to spaceflight. *Medicine and Science in Sports and Exercise*.

[2] Watenpaugh DE, Hargens AR, Fregly MJ, Blatteis CM (1996). The cardiovascular system in microgravity. *Handbook of Physiology*.

[3] Buckey JC, Gaffney FA, Lane LD (1996). Central venous pressure in space. *Journal of Applied Physiology*.

[4] Kirsch KA, Rocker L, Gauer OH (1984). Venous pressure in man during weightlessness. *Science*.

[5] O’Leary DS, Pantalos GM, Sharp MK (1999). Feedback control of mean aortic pressure in a dynamic model of the cardiovascular system. *ASAIO Journal*.

[6] Prisk GK, Guy HJB, Elliott AR, Deutschman RA, West JB (1993). Pulmonary diffusing capacity, capillary blood volume, and cardiac output during sustained microgravity. *Journal of Applied Physiology*.

[7] Fritsch-Yelle JM, Charles JB, Jones MM, Wood ML (1996). Microgravity decreases heart rate and arterial pressure in humans. *Journal of Applied Physiology*.

[8] Gazenko OG, Shulzhenko EB, Turchaninova VF, Egorov AD (1988). Central and regional hemodynamics in prolonged space flights. *Acta Astronautica*.

[9] Shimizu T (1989). Postnatal development of regulatory function of the circulation. *Journal of the Physiological Society of Japan*.

[10] Waki H, Yamasaki M, Katahira K, Katsuda S, Maeda M, Shimizu T (2008). Developmental changes in functional characteristics of aortic baroreceptor afferents in rats. *Experimental Physiology*.

[11] Kasparov S, Paton JFR (1997). Changes in baroreceptor vagal reflex performance in the developing rat. *Pflugers Archiv*.

[12] Dickhout JG, Lee RMKW (1998). Blood pressure and heart rate development in young spontaneously hypertensive rats. *American Journal of Physiology. Heart and Circulatory Physiology*.

[13] Angell-James JE (1971). The responses of aortic arch and right subclavian baroreceptors to changes of non-pulsatile pressure and their modification by hypothermia. *The Journal of Physiology*.

[14] Angell-James JE (1974). Arterial baroreceptor activity in rabbits with experimental atherosclerosis. *Circulation Research*.

[15] Burton AC (1954). Relation of structure to function of the tissues of the wall of blood vessels. *Physiological Reviews*.

[16] Apter JT, Marquez E (1968). Correlation of visco-elastic properties of large arteries with microscopic structure. *Circulation Research*.

[17] Attinger FM (1968). Two-dimensional in-vitro studies of femoral arterial walls of the dog. *Circulation Research*.

[18] Bader H, Hamilton WF (1963). The anatomy and physiology of the vascular wall. *Handbook of Physiology*.

[19] Azuma T, Hasegawa M (1971). A rheological approach to the architecture of arterial walls. *Japanese Journal of Physiology*.

[20] Hasegawa M, Azuma TT Rheological properties of the main vascular system: with special reference to the fine structure of walls.

[21] Shimizu T, Yamasaki M, Nagayama T Changes in the common carotid arterial flow in the rabbit and rat during parabolic flight.

[22] Nagayama T, Katsuda S, Waki H (1997). Changes in femoral arterial flow in the rabbit under conditions of microgravity elicited during parabolic flight. *Japanese Journal of Physiology*.

[23] Waki H, Shimizu T, Katahira K, Nagayama T, Yamasaki M, Katsuda S (2002). Effects of microgravity elicited by parabolic flight on abdominal aortic pressure and heart rate in rats. *Journal of Applied Physiology*.

[24] Yamasaki M, Shimizu T (2002). Effects of the head-down tilt posture on postnatal development of the aortic baroreflex in the rabbit. *Japanese Journal of Physiology*.

[25] Buckey JC, Linnehan RM, Dunlap AW *The Neurolab Spacelab Mission: Neuroscience Research in Space, Results From the STS-90, Neurolab Spacelab Mission*.

[26] Buckey JC, Linnehan RM, Dunlap AW, Buckey JC, Homic JL (2003). Animal care on neurolab. *The Neurolab Spacelab Mission: Neuroscience Research in Space*.

[27] Katsuda S, Waki H, Yamasaki M (2002). Postnatal changes in the rheological properties of the aorta in Sprague-Dawley rats. *Experimental Animals*.

[28] Lawton RW (1955). The thermoelastic behavior of isolated aortic strips of the dog. *Circulation Research*.

[29] McDonald DA (1961). *Blood Flow in Arteries*.

[30] Shimizu T (1999). Development of the aortic baroreflex system under conditions of microgravity. *Journal of Gravitational Physiology*.

[31] Waki H, Katahira K, Yamasaki M (2005). Effects of spaceflight on postnatal development of arterial baroreceptor reflex in rats. *Acta Physiologica Scandinavica*.

[32] Harkness ML, Harkness RD, McDonald DA (1957). The collagen and elastin content of the arterial wall in the dog. *Proceedings of the Royal Society of London B*.

[33] Krafka J (1938). Comparative study of histophysics of the aorta. *American Journal of Physiology*.

[34] Cox TH (1977). Effects of age on the mechanical properties of rat carotid artery. *The American Journal of Physiology—Heart and Circulatory Physiology*.

[35] Cox RH (1978). Comparison of carotid artery mechanisms in the rat, rabbit, and dog. *The American Journal of Physiology—Heart and Circulatory Physiology*.

[36] Cox RH (1978). Regional variation of series elasticity in canine arterial smooth muscles. *The American Journal of Physiology*.

[37] Cox RH (1979). Regional, species, and age related variations in the mechanical properties of arteries. *Biorheology*.

[38] Hasegawa M, Watanabe Y (1988). Rheological properties of the thoracic aorta in normal and WHHL rabbits. *Biorheology*.

[39] Roach M, Burton AC (1957). The reason for the shape of the distensibility curves of arteries. *Canadian Journal of Biochemistry and Physiology*.

[40] Roach MR, Burton AC (1959). The effect of age on the elasticity of human iliac artery. *Canadian Journal of Biochemistry and Physiology*.

[41] Walton KD, Kalb RG, Luis JD, Buckey JC, Homick JL (2003). Motor system development depends on experience: a microgravity study of rats. *Neurolab Spacelab Mission: Neuroscience Research in Space, Results From the STS-90, Neurolab Spacelab Mission*.

[42] Adams GR, Haddad F, Baldwin KM, Buckey JC, Homick JL (2003). Gravity plays an important role in muscle development and the differentiation of contractile protein phenotype. *Neurolab Spacelab Mission: Neuroscience Research in Space, Results From the STS-90, Neurolab Spacelab Mission*.

[43] Oishi H, Shimizu T, Katahira K (1998). Effects of microgravity to the mechanical changes in the rat aorta. *Fy1997 Ground-based Research Announcement for Space Utilization Research Report*.

[44] Stein O, Eisenberg S, Stein Y (1971). Morphologic and biochemical changes in smooth muscle cells of aortas in growth-restricted rats. *Laboratory Investigation*.

[45] Adams GR, McCue SA, Bodell PW, Zeng M, Baldwin KM (2000). Effects of spaceflight and thyroid deficiency on hindlimb development. I. Muscle mass and IGF-I expression. *Journal of Applied Physiology*.

[46] Yamasaki M, Shimizu T, Katahira K (2004). Spaceflight alters the fiber composition of the aortic nerve in the developing rat. *Neuroscience*.

